# Clinical Significance of Diffusely Increased Uptake of ^68^Ga-FAPI in Thyroid Gland

**DOI:** 10.3389/fmed.2021.782231

**Published:** 2021-11-23

**Authors:** Huipan Liu, Xiao Yang, Lin Liu, Lei Lei, Li Wang, Yue Chen

**Affiliations:** ^1^Department of Nuclear Medicine, The Affiliated Hospital of Southwest Medical University, Luzhou, China; ^2^Nuclear Medicine and Molecular Imaging Key Laboratory of Sichuan Province, Luzhou, China; ^3^Academician (Expert) Workstation of Sichuan Province, Luzhou, China

**Keywords:** endocrinology, PET/CT, ^68^Ga-FAPI, diffuse thyroid uptake, thyroiditis

## Abstract

**Purpose:** To determine the clinical significance of diffuse uptake of ^68^Ga-FAPI in the thyroid.

**Methods:** From January 2020 to September 2021, all subjects with diffuse thyroid uptake in ^68^Ga-FAPI PET/CT were investigated in our hospital, and compared with the age and sex matched control group. The ^68^Ga-FAPI uptake in the thyroid gland was analyzed semi-quantitatively using the maximum standardized uptake value (SUVmax), and regression analysis was used to analyze the correlation between available serum thyroid stimulating hormone (TSH) and thyroid peroxidase antibody (TPOAb).

**Results:** Among 815 subjects, 39 subjects were found diffuse FAPI uptake in thyroid gland; 11 subjects refused further examination; a total of 28 subjects were included in the analysis, and 27 subjects were diagnosed with chronic thyroiditis (including 20 subjects with Hashimoto's thyroiditis), 3 subjects with Grave's disease, 3 subjects with only serum TSH elevated, and 1 subject with malignant of thyroid and thyroiditis. The SUVmax of 27 subjects with thyroiditis was 5.75 ± 5.45. No significant correlation was found between the SUVmax and the level of serum TSH (*P* = 0.389) or TPOAb (*P* = 0.426).

**Conclusion:** The incidentally discovered diffusely increased ^68^Ga-FAPI uptake in the thyroid gland is mostly related to chronic lymphocytic (Hashimoto's) thyroiditis. ^68^Ga-FAPI uptake level correlated neither with the degree of hypothyroidism nor with the titer of TPOAb. In addition, immune-related thyroiditis with immune checkpoint inhibitors may be accidentally found on ^68^Ga-FAPI, which may be helpful in facilitate timely intervention.

## Introduction

^68^Ga-FAPI is a novel tumor-targeting agent, as fibroblast activation protein is overexpressed in cancer-associated fibroblasts (1–3). Existing research has shown that FAPI revealed favorable pharmacokinetics and biodistribution *in vivo* and a clear delineation of primary tumors and their metastases (4–7). With the increasing use of ^68^Ga-FAPI in clinical trials, accurate interpretation of unexpected findings remains a challenge, because the impact of accidental findings on patient management may be significant.

Chronic thyroiditis is confirmed to be thyroid degeneration and fibrotic changes. Because of its hard or rubber-like texture and nodules on the surface, it is easy to be mistaken for malignant tumors. Previously, we encountered a patient with diffuse FAPI uptake in thyroid, and the findings suggested a thyroid tumor. The diagnosis of thyroid tumor was also based on ultrasound. However, histopathological examination revealed chronic thyroiditis (Hashimoto's) in the nodule and surrounded by thyroid tissue (8). In addition, we also encountered a patient with diffuse FAPI uptake in the thyroid. At first, it was considered as chronic thyroiditis, but finally diagnosed as thyroid lymphoma (diffuse large B-cell lymphoma) (9). Therefore, when judging ^68^Ga-FAPI PET/CT images, diffuse FAPI uptake in the thyroid needs to be identified.

In this study, we prospectively analyzed ^68^Ga-FAPI PET/CT images to determine the frequency and clinical significance of diffuse FAPI uptake in the thyroid.

## Subjects and Methods

Patients with solid tumors or suspected tumors were enrolled in a ^68^Ga-FAPI PET/CT tumor clinical trial (ChiCTR2100044131) approved by the institutional review committee of our hospital, and the patients with diffuse high ^68^Ga-FAPI uptake in the thyroid gland from January 2020 to September 2021 were included in this studied. Patients with focal thyroid uptake and those having a history of thyroid cancer were excluded.

Patients were prospectively enrolled at the time each PET examination was done during the enrollment period. Patients who had more than one examination during the study period were counted only once. Distinctly increased ^68^Ga-FAPI uptake in the thyroid, resulting in visualization of both thyroid lobes on the 3-dimensional maximum-intensity-projection images, was used as the sole criterion for inclusion. Only the first PET examination demonstrating this finding was studied for each patient. Thyroid uptake was always then confirmed on axial PET/CT fused images. Inclusion was decided by a consensus of 2 experienced PET/CT readers.

Uptake in the thyroid was measured in a semiquantitative manner as maximum standardized uptake value (SUVmax) corrected for body weight. A circular region of interest with a fixed diameter of 1.5 cm was placed over the region of highest intensity in each lobe of the thyroid, and the uptake was automatically quantified as SUVmax, and calculated target/background rates (TBR) using liver SUVmean. The higher of the 2 values between the thyroid lobes was used in the statistics.

Patients included in the study were followed for any subsequent clinical or laboratory evaluation of thyroid status. Clinical information was also reviewed retrospectively to determine whether there had been a previous diagnosis of thyroid disease; ultrasound studies of the thyroid or neck; measurements of thyroid function tests (FT3, FT4, TSH, TgAb, TPOAb); or use of thyroid hormone medication. This review was done through a detailed study of the medical records.

A control group of patients with no thyroid uptake was also studied. Age- and sex-matched control subjects were randomly selected from the group of individuals without diffuse thyroidal FAPI uptake. The control group consisted of the same number of patients as those found to have diffuse thyroid uptake. The charts of these patients were studied for evidence of any thyroid disease.

Finally, from those patients with diffuse thyroid uptake and no prior history of thyroid disease, we selected only those having thyroid function tests (FT3, FT4, TSH, TgAb, TPOAb) measured within 8 week after the PET study.

### ^68^Ga-FAPI PET/CT

We purchased the precursor FAPI-04 from MCE (MedChemExpress, USA), with a purity of 98% and a quality of 872.91. The ^68^Ga-FAPI labeling was carried out according to the method described previously (10). The radiochemical purity of ^68^Ga-FAPI exceeded 95%. The sterility test was performed by the radiochemical equipment of the Department of Nuclear Medicine, the Affiliated Hospital of Southwest Medical University. The final product was sterile and meet all the required standards of our institution before use.

The intravenous radiotracer dose was 1.85–2.59 MBq/kg, and imaging was performed 40–60 min after radiotracer injection (United Imaging UMI 780 PET/CT). All subjects were required to urinate as much as possible for imaging preparations, which reduced the influence of the residual radiotracer in the renal. The scope of the whole-body inspection was from the base of the skull to the base of the thigh, using 5 to 6 beds (3 min/bed). The matrix was 128 × 128, FOV 600 mm, the PET layer thickness was 3 mm, and all PET images were reconstructed iteratively (OSEM). After the reconstruction was completed, the post-processing and fusion software of United Imaging was used for image analysis.

### Laboratory Tests

In each subject with diffuse FAPI uptake in the thyroid, serum thyroid hormones, including free thyroxine (FT3, reference range, 1.80–3.80 pg/ml; FT4, reference range, 0.78–1.86 ng/dl), TSH (reference range, 0.38–5.57 mIU/L), TgAb (reference range, 0.0–14.58 IU/ml), and TPOAb (reference range, 0.0–2.6 IU/ml) levels were measured within 8 weeks.

### Ultrasonography

All subjects underwent ultrasound examination. The sonographer did not know the results of the PET/CT scan. In subjects with positive thyroid FAPI uptake, after the first PET/CT examination, ultrasound scan was performed to check the following abnormalities: (I) abnormal size (atrophy or goiter), (II) irregular surface, (III) the internal echo was low, or (IV) the internal echo pattern was uneven.

### Statistical Analyses

All statistical analyses were conducted using SPSS 22.0 software. The study and control groups were compared with the Student *t*-test for unpaired data. Measures of FAPI uptake and laboratory test results were compared by using the Fisher exact test.

## Results

Diffuse FAPI uptake of thyroid was found in 39 of 815 (4.8%) subjects between January 2020 and September 2021 [(man accounted for 2.6% (21/815) and woman accounted for 2.2% (18/815)], and their mean age was 53.3 ± 10.9 y.

**Figure 1:**
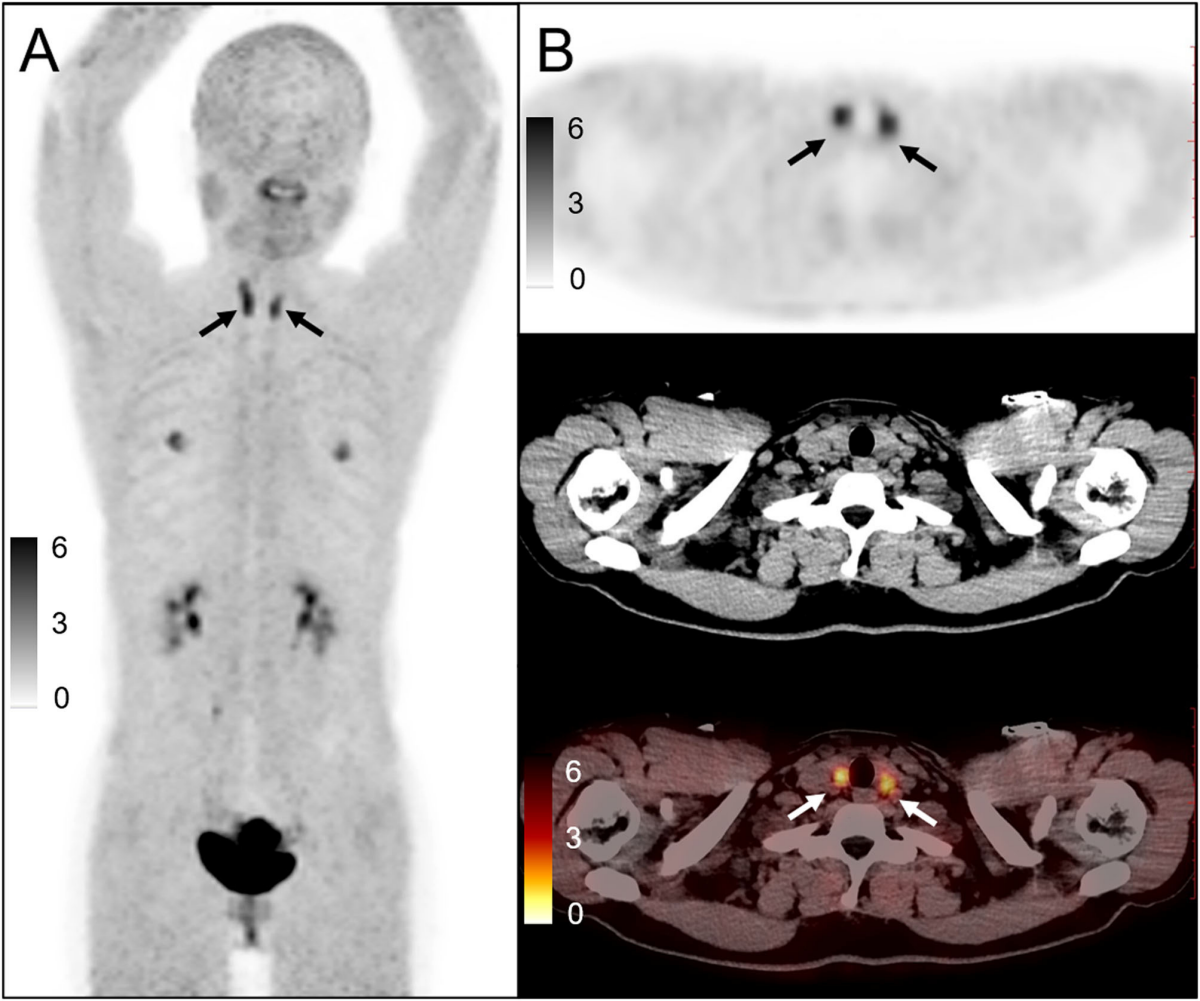
A 32-year-old woman underwent ^68^Ga-FAPI PET/CT for rectal cancer after surgery [**(A)**: maximum intensity projection image; **(B)**: upper, PET, middle, CT, lower, PET/CT image]. The uptake of thyroid ^68^Ga-FAPI increased diffusedly, and the SUVmax was about 4.6, and TBR was 7.54. CT showed a decrease in density. Ultrasound examination showed that the echo of the thyroid gland was reduced and the blood flow was uneven. A fine needle aspiration biopsy of the thyroid revealed chronic thyroiditis. Thyroid function test revealed abnormal TSH and thyroid antibody levels (TSH: 16.117, reference range, 0.38–5.57 mIU/L; FT3:2.07, reference range, 1.80–3.80 pg/ml; FT4: 0.86, reference range, 0.78–1.86 ng/dl; TPOAb: 872.18, reference range, 0.0–2.6 IU/ml; TgAb: 620.47, reference range, 0.0–14.58 IU/ml), conforming the diagnosis of lymphocytic thyroiditis.

Eleven of these patients were denied access to their data for research purposes. Therefore, a total of 28 subjects were included in the analysis (Table 1). The clinical data of the study subjects were retrospectively analyzed. No prior history of thyroid disease was found in the 28 subjects. Diffuse FAPI uptake in the thyroid gland was shown in Figure 1. CT showed that the density of 21 subjects was unevenly reduced.

**Table 1:** Characteristics of subjects with diffuse thyroidal FAPI.

**Subjects**	SUVmax^*^	**TBR^$^**	**Density**	**Echo pattern**	**Surface**	**Echo level**	**Size**	**T3**	**T4**	**TSH**	**TgAb**	**TPOAb**
1	3.9	5.95	Reduced	Inhomogeneous	Irregular	Hypoecho	Large	2.01	1.12	4.173	91.82	15.69
2	3.0	4.91	Normal	Normal	Regular	Normal	Normal	7.14	1.26	4.504	<0.12	<0.05
3	4.6	7.95	Reduced	Inhomogeneous	Regular	Hypoecho	Normal	2.07	0.86	16.117	620.47	872.18
4	6.9	15	Reduced	Inhomogeneous	Irregular	Hypoecho	Large	2.29	0.86	4.368	714.45	61.99
5	3.9	7.01	Normal	Inhomogeneous	Regular	Normal	Normal	2.02	1.22	8.911	<0.12	<0.05
6	4.1	7.69	Reduced	Normal	Regular	Normal	Normal	2.86	1.32	6.017	<0.12	<0.05
7	3.2	7.71	Reduced	Inhomogeneous	Regular	Hypoecho	Normal	1.93	1.1	1.601	59.38	262.8
8	4.2	7.63	Reduced	Inhomogeneous	Regular	Hypoecho	Normal	2.46	0.82	15.901	15.74	11.85
9	4.5	5.13	Reduced	Inhomogeneous	Irregular	Hypoecho	Large	17.11	4.51	<0.01	252.69	>1,000
10	2.8	5.42	Reduced	Inhomogeneous	Irregular	Hypoecho	Large	5.38	3.11	<0.01	109.15	32.19
11	7.2	12.67	Reduced	Inhomogeneous	Irregular	Hypoecho	Large	>25	>8	<0.01	>2,000	>1,000
12	3.9	7.35	Normal	Normal	Regular	Normal	Normal	2.43	1.19	6.977	<0.12	<0.05
13	7.5	9.23	Reduced	Inhomogeneous	Regular	Hypoecho	Small	1.44	1.57	4.281	865.1	985.85
14	8	16.26	Reduced	Inhomogeneous	Regular	Normal	Small	2.09	0.9	2.938	7.59	152.82
15	4.6	8.98	Reduced	Inhomogeneous	Irregular	Hypoecho	Large	2.64	1.18	1.501	205.68	765.23
16	4.6	9.09	Normal	Inhomogeneous	Regular	Normal	Normal	2.29	1.22	3.145	>2,000	<0.05
17	6.1	7.45	Normal	Inhomogeneous	Regular	Normal	Normal	2.86	1.18	13.193	12.06	<0.05
18	3.4	6.03	Normal	Inhomogeneous	Regular	Normal	Normal	2.48	1.11	1.54	<0.12	<0.05
19	3.3	6.91	Normal	Inhomogeneous	Irregular	Normal	Small	1.29	1.23	2.64	<0.12	<0.05
20	4.9	8.19	Reduced	Inhomogeneous	Regular	Normal	Small	2.23	0.54	31.895	350.81	<0.05
21	3.6	6.74	Reduced	Inhomogeneous	Regular	Normal	Normal	2.53	1.32	3.99	59.62	<0.05
22	3.3	6.32	Reduced	Inhomogeneous	Irregular	Normal	Small	2.36	1.01	0.977	38.82	6.88
23	6.3	8.67	Reduced	Inhomogeneous	Irregular	Hypoecho	Large	2.53	1.32	9.36	36.82	8.31
24	6.9	15.71	Reduced	Inhomogeneous	Irregular	Hypoecho	Large	1.44	0.89	2.812	264.59	>1,000
25	4.1	11.14	Reduced	Inhomogeneous	Irregular	Hypoecho	Normal	2.51	1.28	4.243	646.07	<0.05
26	3.9	7.78 8	Reduced	Inhomogeneous	Irregular	Hypoecho	Normal	2.6	1.5	4.1	106.23	<0.05
27	32	38.55	Reduced	Inhomogeneous	Irregular	Hypoecho	Large	3.4	1.67	38.78	690.35	>1,000
28	3.6	6.314	Reduced	Inhomogeneous	Irregular	Hypoecho	Small	2.6	1.45	8.99	<0.12	<0.05

* *SUVmax of Thyroid*.

$ *TBR (target/background rates) = SUVmax of Thyroid/SUVmean of liver;the normal reference range of FT3 was 1.80–3.80 pg/ml, FT4 was 0.78–1.86 ng/dl, TSH was 0.38–5.57 mIU/L, TgAb was 0.0–14.58 IU/ml, and TPOAb was 0.0–2.6 IU/ml*.

### Biochemical Findings

Nine subjects had increased levels of TSH [median 9.36 (range 6.017–38.78)], and levels of FT3 [median 2.53 (range 2.02–3.4)] and FT4 [median 1.22 (range 2.82–1.67)] were normal. These findings met the criteria for subclinical hypothyroidism; three subjects had elevated levels of FT3 [median 17.11 (range NA)] and FT4 [median 4.51 (range NA)], and decreased the levels of TSH (<0.01), which met the diagnostic criteria for Grave's disease. Twenty subjects were positive for anti-thyroid antibodies [TPOAb (median 252.69) and/or TgAb (median 262.8)]; one subject only had increased level of FT3 (7.14); one subject only had decreased level of FT3 (1.29).

### Ultrasound Findings

Twenty-five subjects showed that the thyroid blood flow signal was uneven in ultrasonography; 16 subjects had hypoechoic thyroid; 14 subjects had irregular thyroid edges; nine subjects had enlarged thyroid glands, which were obviously enlarged on palpation; six subjects had reduced thyroid volume.

### Clinicopathological Findings

Twenty-seven subjects were diagnosed with chronic thyroiditis (including 20 subjects with Hashimoto's thyroiditis), of which 5 subjects of chronic (Hashimoto's) thyroiditis were diagnosed by histopathological results; eight subjects were diagnosed with hypothyroidism or subclinical hypothyroidism; three subjects were diagnosed with Grave's disease; one subjects had normal thyroid function, but thyroid blood flow signal was uneven, which was consistent with chronic thyroiditis; three subjects had only elevated serum TSH; one subject was pathologically confirmed to be thyroid lymphoma with thyroiditis. Among the 28 subjects, six subjects underwent two or more PET/CT scans within 1 year, and four subjects were observed with similar diffuse FAPI uptake.

### ^68^Ga-FAPI Findings

The median SUVmax of 28 subjects was 4.15 (range 2.8–32), and the median serum level of TSH was 4.208 (range 0.01–31.89). No significant correlation was found between the serum TSH and FAPI uptake (*P* = 0.389). Similarly, the median serum level of TPOAb was 7.595 (range 0.05–1,000), and there was no significant correlation between serum TPOAb and FAPI uptake (*P* = 0.426). In the 27 subjects with thyroiditis, the SUVmax of thyroid was 5.75 ± 5.45.

**Figure 2:**
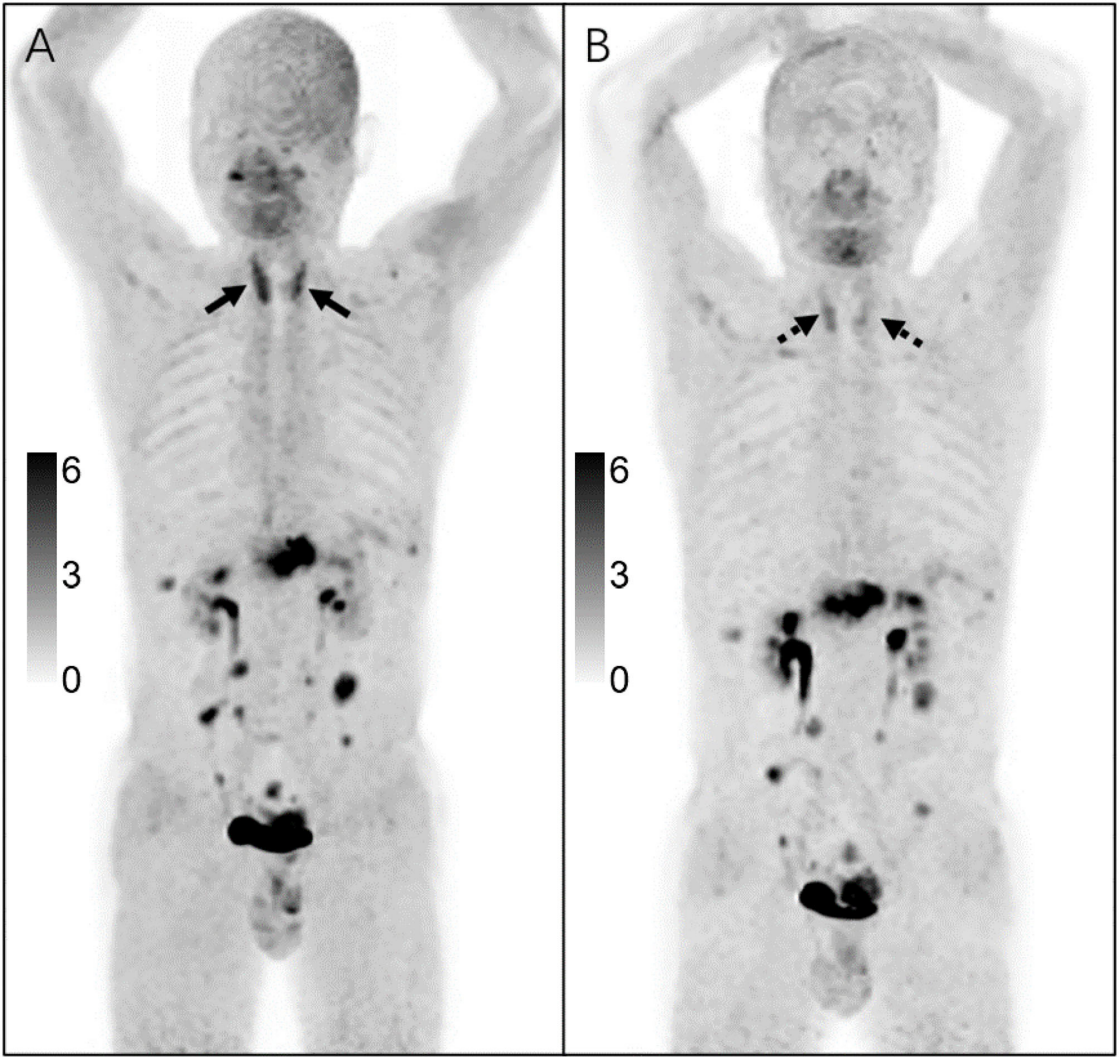
A 58-year-old man was diagnosed with peritoneal metastasis due to post-operative re-examination of pancreatic cancer. After receiving Carrelizumab immunosuppressive therapy, ^68^Ga-FAPI PET/CT was performed [**(A)**: after 1 cycle; **(B)**: after 4 cycles]. After the first cycle, the thyroid showed diffuse increased FAPI uptake with SUVmax of 6.1, and TBR 7.46; blood analysis showed FT3 2.86, FT4 1.18, TSH 13.193, TgAb 12.06, and TPOAb <0.05; after the fourth cycle, the thyroid also showed diffuse increased FAPI uptake with SUVmax of 3.9, and TBR 6.26; blood analysis showed FT3 2.49, FT4 1.02, TSH 7.096, TgAb <0.12, and TPOAb <0.05; Finally, a diagnosis of immune-related thyroiditis was made.

In addition, before the ^68^Ga-FAPI PET/CT examination, three subjects received immunotherapy, the SUVmax of thyroid was 4.33 ± 1.70, of which two subjects were undergoing immunosuppressive therapy, and one subject ended immunosuppressive therapy before 2 months (Table 2). Subject No. 2 had only elevated T3 after FAPI examination and was asymptomatic. However, with 3 months of follow-up, it was found that the subject was diagnosed with subclinical hypothyroidism (TSH: 6.8 mIU/ml). Therefore, three subjects were diagnosed with immune-related thyroiditis (Figure 2).

**Table 2:** Status of subjects receiving immunosuppressive therapy.

**Subjects number**	**Drug**	**Cycle(s)**	Interval between PET/CT month(s)^*^	**SUVmax of thyroid**	**TBR**
2	Rituximab	4	2	3	4.9
9	Navuliumab	3	1	4.2	7.64
17	Carrelizumab	1	1	6.1	7.46

* *The interval between PET/CT examination and the end of the previous cycle of treatment*.

### Control Subjects

Twenty-eight subjects in the control group had no FAPI uptake in the thyroid gland (Table 3). In subsequent thyroid function tests, 3 subjects had decreased FT3 [median 1.65 (range 1.63–1.72)], no antibody-positive subjects were found, and 2 subjects had abnormal thyroid blood flow signals. The positive rate of thyroid function tests and abnormal ultrasound in the control group was lower than that in the test group, and the difference was statistically significant (*P* <0.001).

**Table 3:** Comparison of the diffuse thyroidal uptake group and the control group with regards to the findings on ultrasound, thyroid function tests, and ^68^Ga-FAPI PET/CT.

**Indicators**	**US abnormalities cases**	**Thyroid function tests abnormalities cases**	**SUVmax of thyroid**	**SUVmean of liver**	**TBR**
Test group	26	27	5.75 ± 5.45	0.58 ± 0.12	9.56 ± 6.44
Control group	2	3	1.36 ± 0.26	0.52 ± 0.05	2.68 ± 0.46
*P*-value	<0.001	<0.001	<0.001	0.222	<0.001

### Adverse Effects

There were no adverse or clinically detectable pharmacologic effects in any of the subjects.

## Discussion

The application of ^68^Ga-FAPI in tumors is gradually being reported, and the wide application of PET/CT in clinical practice emphasizes the importance to clarify the accidental findings. To our knowledge, the current literatures do not mention the clinical significance of ^68^Ga-FAPI uptake levels in the thyroid.

Our study found that 39 of 815 (4.8%) subjects had diffuse FAPI uptake in the thyroid. Among the 28 verified subjects, 27 subjects had thyroiditis, and 3 subjects had Grave's disease. Only three subjects had elevated serum level of TSH, and one subject was thyroid lymphoma.

Chronic thyroiditis is an autoimmune disease that is prevalent among elderly women. This is the main cause of hypothyroidism in the elderly. Hormonal abnormalities appear before symptoms and signs appear. As diagnostic procedures improve, some researchers believe that the frequency of this disease may be increasing. Our study showed that only one subject had significant hypothyroidism, and eight subjects were considered to have subclinical hypothyroidism. In addition, immunosuppressive therapy may also cause immune-related thyroiditis (11). In our study, it was found that 3 subjects had thyroiditis caused by immunosuppressive therapy, and the thyroid uptake was relatively low, which may be related to the shorter PET/CT scan interval after treatment. Immune checkpoint inhibitors are cancer therapies that provide impressive clinical benefit in many advanced malignancies. Immune-related thyroiditis with immune checkpoint inhibitors is a common consequence, while its natural course and management recommendations are not well-characterized (12). Some researchers suggested that many patients have a subclinical thyroiditis that does not warrant any specific treatment (13). Existing guidelines recommend that thyroid hormone levels be checked at least every 6 months for patients with drug-induced thyroiditis (14). However, study has shown that checking at 6-month intervals would miss not just the thyrotoxic phase but also the onset of hypothyroidism in patients receiving immune checkpoint inhibitor, potentially leading to worsening patient morbidity associated with untreated hypothyroidism (13). Our results indicate that immune-related thyroiditis with immune checkpoint inhibitors may be accidentally found on ^68^Ga-FAPI, which may be helpful in facilitate timely intervention.

In this study, the median SUVmax of the thyroid was 4.15 (range: 2.8–32.0), and SUVmax of the control group of subjects was 1.36 ± 0.26. Considering the previously reported average SUVmean of the normal control group thyroid gland is 2.3 ± 1.0, our value seems to be relatively low, which may be related to the difference in acquisition equipment and acquisition parameters (15). We did not find any significant correlation between the level of serum TSH and FAPI uptake in the thyroid, which suggests that even mild or low FAPI uptake in the thyroid should not be ignored. Meanwhile, there was no significant correlation between the level of serum TPOAb and FAPI uptake in the thyroid.

The mechanism of ^68^Ga-FAPI uptake by the thyroid remains unclear. Lymphocyte and fibroblast infiltration are the histological features of chronic thyroiditis (16). The uptake of ^68^Ga-FAPI in thyroiditis has been reported, and the reason may be related to inflammation-related fibroblasts (8, 17). In addition, the hyperplasia of fibrous tissue in chronic thyroiditis may also be the reason for the increased uptake of FAPI. One subject with lymphoma had an increased uptake of ^68^Ga-FAPI, which was thought to be related to tumor-associated fibroblasts (9).

A potential limitation of the current study is that the number of patients is relatively small, and no definitive conclusions can be made. Thus, larger patient populations are needed to further study. In addition, ^68^Ga-FAPI is mostly performed on tumor subjects, and most non-tumor subjects are not included in the analysis. Therefore, the sample size needs to be expanded to analyze normal subjects. Furthermore, there are certain limitations in visually performing SUVmax analysis. Meanwhile, it should be noted that 11 subjects with diffuse FAPI uptake in the thyroid refused to be further examined, and the specific reasons for their uptake are still unknown. Moreover, focal uptake of FAPI in thyroid gland were excluded in our study, and the comparison of diffuse and focal uptake needs further study, which might be one of the potential uses of FAPI for thyroid diseases. Excluding patients with thyroid cancer may also create a selection bias.

## Conclusion

The incidentally discovered diffusely increased ^68^Ga-FAPI uptake in the thyroid gland is mostly related to chronic lymphocytic (Hashimoto's) thyroiditis. ^68^Ga-FAPI uptake level correlated neither with the degree of hypothyroidism nor with the titer of TPOAb. In addition, immune-related thyroiditis with immune checkpoint inhibitors may be accidentally found on ^68^Ga-FAPI, which may be helpful in facilitate timely intervention. The mechanism of the diffusely increased ^68^Ga-FAPI uptake in the thyroid needs to be further analyzed.

## Data Availability Statement

The original contributions presented in the study are included in the article/supplementary material, further inquiries can be directed to the corresponding author/s.

## Ethics Statement

The studies involving human participants were reviewed and approved by the Ethics Committee of the Affiliated Hospital of Southwest Medical University (AHSWMU-2020-035). The patients/participants provided their written informed consent to participate in this study.

## Author Contributions

HL and XY drafted the manuscript. LLi acquired the PET/CT images. LLe, LW, and YC provided critical review of the manuscript for key intellectual content. HL and YC are the guarantors, and as such, had full access to the data and take responsibility for its integrity and accuracy. HL conducted statistical analyses. All authors conceived and designed the study, interpreted the findings, and approved the final manuscript.

## Conflict of Interest

The authors declare that the research was conducted in the absence of any commercial or financial relationships that could be construed as a potential conflict of interest.

## Publisher's Note

All claims expressed in this article are solely those of the authors and do not necessarily represent those of their affiliated organizations, or those of the publisher, the editors and the reviewers. Any product that may be evaluated in this article, or claim that may be made by its manufacturer, is not guaranteed or endorsed by the publisher.
